# Ring finger protein 39 genetic variants associate with HIV-1 plasma viral loads and its replication in cell culture

**DOI:** 10.1186/2045-3701-4-40

**Published:** 2014-08-05

**Authors:** Ying-Ju Lin, Chia-Yen Chen, Kuan-Teh Jeang, Xiang Liu, Jen-Hsien Wang, Chien-Hui Hung, Hsinyi Tsang, Ting-Hsu Lin, Chiu-Chu Liao, Shao-Mei Huang, Cheng-Wen Lin, Mao-Wang Ho, Wen-Kuei Chien, Jin-Hua Chen, Tsung-Jung Ho, Fuu-Jen Tsai

**Affiliations:** 1 Genetic Center, Department of Medical Research, China Medical University Hospital, Taichung, Taiwan; 2 School of Chinese Medicine, China Medical University, Taichung, Taiwan; 3 Viral Biochemistry Section, Laboratory of Molecular Microbiology, National Institute of Allergy and Infectious Diseases, National Institutes of Health, Bethesda, Maryland, USA; 4 Molecular Virology Section, Laboratory of Molecular Microbiology, National Institute of Allergy and Infectious Diseases, National Institutes of Health, Bethesda, Maryland, USA; 5 Section of Infectious Diseases, Department of Internal Medicine, China Medical University Hospital, Taichung, Taiwan; 6 Graduate Institute of Clinical Medical Science, Chang-Gung University, Chiayi, Taiwan; 7 The Laboratory of Molecular Immunogenetics, National Institute of Allergy and Infectious Diseases, National Institutes of Health, Bethesda, MD, USA; 8 Department of Medical Laboratory Science and Biotechnology, China Medical University, Taichung, Taiwan; 9 Biostatistics Center, China Medical University, Taichung, Taiwan; 10 Biostatistics Center, Taipei Medical University, Taipei, Taiwan; 11 Division of Chinese Medicine, China Medical University Beigang Hospital, Yunlin County, Taiwan; 12 Division of Chinese Medicine, Tainan Municipal An-Nan Hospital -China Medical University, Tainan, Taiwan; 13 Department of Biotechnology, Asia University, Taichung, Taiwan

**Keywords:** HIV-1 viral load, *RNF39*, Single nucleotide polymorphism, Viral replication

## Abstract

**Background:**

The human immunodeficiency virus (HIV-1) exploits host proteins to complete its life cycle. Genome-wide siRNA approaches suggested that host proteins affect HIV-1 replication. However, the results barely overlapped. RING finger protein 39 *(RNF39)* has been identified from genome-wide association studies. However, its function during HIV-1 replication remains unclear.

**Methods and results:**

We investigated the relationship between common *RNF39* genetic variants and HIV-1 viral loads. The effect of RNF39 protein knockdown or overexpression on HIV-1 replication was then investigated in different cell lines. Two genetic variants were associated with HIV-1 viral loads. Patients with the ht1-GG/GG haplotype presented lower RNF39 expression levels and lower HIV-1 viral load. RNF39 knockdown inhibited HIV-1 expression.

**Conclusions:**

RNF39 protein may be involved in HIV-1 replication as observed in genetic studies on patients with HIV-1 and in *in vitro* cell cultures.

## Background

The human immunodeficiency virus (HIV-1) genome encodes for 15 viral proteins and exploits host cellular proteins to complete its life cycle [1,2], which includes viral entry, uncoating, reverse transcription, nuclear import, integration, transcription, translation, viral assembly, and virus budding [3-6]. Because of the complicated nature of the viral life cycle and restricted encoded viral proteins, the function of host cellular proteins associated with virus replication remains to be confirmed. Genome-wide siRNA screening approaches have been conducted to identify host cellular proteins that affect HIV-1 replication [7-10]. However, little overlapping was observed between these screening studies. This may be due to diverse experimental conditions, including virus strains, cell line usages, the delivery efficiency of siRNAs or shRNA libraries, and the analysis of RNAi datasets. Therefore, functional characterization of interesting genes and their genetic-clinical correlations in patients with HIV-1 are essential to elucidate the role of host cellular proteins in HIV-1 replication.

Previous genome-wide association studies have mapped to a region close to the *ZNRD1* (zinc ribbon domain-containing 1) and RING finger protein 39 *(RNF39)* associated with HIV-1 disease progression [11,12]. We have also showed that *ZNRD1* genetic variants contribute to HIV-1 clinical course in the Han Chinese population in Taiwan and that *ZNRD1* RNA interference-mediated silencing inhibited HIV-1 replication in Jurkat cells [13]. *RNF39* encodes a protein containing a specialized type of SPRY domain in its C-terminal that determines viral specificity and restriction potency [14]. It is located within the major histocompatibility complex class I region on chromosome 6. However, its biological function during HIV-1 replication remains unclear.

In this study, we conducted a genetic association study between *RNF39* common genetic variants and HIV-1 viral loads in a Han Chinese cohort in Taiwan and evaluated the role of RNF39 in HIV-1 replication. Our results suggest that *RNF39* common genetic variants associate with HIV-1 plasma viral loads and may be required for HIV-1 replication.

## Results

### *RNF39* genetic variants, rs3807032, rs3807033, and related haplotypes, associate with HIV-1 viral load levels

To further investigate the role of *RNF39* genetic variants in virus replication in patients infected by HIV-1, plasma HIV-1 viral load levels and *RNF39* genotype data were collected from a cohort of Han Chinese patients infected with HIV-1 in Taiwan (Tables 1 and 2). The clinical characteristics are summarized in Table 1. Three-hundred and three patients infected with HIV-1 (87.3% of male patients) were included in this study. The mean plasma HIV-1 viral load (before antiretroviral therapy) was 4.0 (2.6–6.4) log_10_ copies/mL. The association between the mean of all available plasma HIV-1 viral load measurements and *RNF39* single nucleotide polymorphism (SNP) genotypes is presented in Table 2. There were significant differences in mean HIV-1 viral load levels among infected patients with *RNF39* SNP genotypes rs3807032 and rs3807033. For patients presenting the SNP rs3807032 GG and CG + CC genotypes, the mean log_10_ HIV-1 viral load was 3.9 (2.6–6.4) and 4.2 (2.6–5.8), respectively (*p* = 0.002). Similar HIV-1 viral loads were also observed for patients presenting the rs3807033 GG and AG + AA genotypes, respectively (*p* = 0.002). Next, the mean HIV-1 viral loads were compared among *RNF39* haplotypes (Table 3). A significant difference in viral loads was observed (*p* = 0.006). Patients with the *RNF39* ht1-GG/GG haplotype presented lower viral loads than patients with the ht2-GG/CA and ht3-CA/CA haplotypes (*p* = 0.006).

**Table 1:** Baseline characteristics of patients with HIV-1 in the Han Chinese population in Taiwan

**Variable**	**Taiwanese HIV-1 infected patients**
No. of participants	303
Male,%	87.3
Mean age at HIV-1 positive (range), years^a^	37.0 (20.6–78.7)
Mean plasma HIV-1 viral load (interquartile range), log_10_ copies/mL^b^	4.0 (2.6–6.4)

^a^The age at HIV-1 antibody positive means the age of the patient when he/she was examined with the earliest HIV-1 antibody positive result. The HIV-1 antibody positive results were obtained from the department of medical and laboratory examination database at our hospital.

^b^The HIV-1 viral load was measured in peripheral blood when the patient was examined with the earliest HIV-1 antibody positive result. Any measurements taken after the initiation of antiretroviral therapy were not used in any analyses.

**Table 2:** **Effects of *RNF39 *** **gene SNPs on plasma HIV-1 viral load in the Han Chinese Population in Taiwan**

**Gene**	**Location**	**Position**	**SNP**		**Genotype**	**Taiwanese HIV-1 infected patients**		
						**Genotype frequency (No (%))**	**HIV-1 Viral Load (log10 copies/mL, mean (interquartile range))**^**a**^	***p*-value**^**b**^
*RNF39*	5′ near gene	30,043,779	SNP1	rs3807032	GG*	219 (72.3)	3.9 (2.6–6.4)	0.002*
					CG + CC	74 (24.4) + 10 (3.3)	4.2 (2.6–5.8)	
	5′ near gene	30,043,955	SNP2	rs3807033	GG*	220 (72.6)	3.9 (2.6–6.4)	0.002*
					AG + AA	73 (24.1) + 10 (3.3)	4.2 (2.6–5.8)	
	5′ near gene	30,044,388	SNP3	rs3132682	CC*	95 (31.4)	3.9 (2.6–5.3)	0.299
					CG + GG	127 (41.9) + 81 (26.7)	4.0 (2.6–6.4)	
	5′ near gene	30,044,827	SNP4	rs3807035	GG*	202 (66.7)	3.9 (2.6–6.4)	0.034
					AG + AA	89 (29.4) + 12 (4.0)	4.1 (2.6–5.8)	
	5′ near gene	30,044,914	SNP5	rs3807036	GG*	283 (93.4)	4.0 (2.6–6.4)	0.199
					AG + AA	20 (6.6) + 0 (0.0)	3.7 (2.6–4.8)	
	5′ near gene	30,045,199	SNP6	rs1150735	GG*	159 (52.5)	3.9 (2.6–5.3)	0.837
					AG + AA	114 (37.6) + 30 (9.9)	4.0 (2.6–6.4)	

The asterisk and bold, emphasizing statistical significance at *p* < 0.0083 (0.05/6).

^a^The HIV-1 viral load was measured in the peripheral blood when the patient was examined with the earliest HIV-1 antibody positive result. Any measurements taken after the initiation of antiretroviral therapy were not used in any analyses.

^b^*p*-values were obtained using the unpaired Student’s *t*-test.

**Table 3:** **Effects of *RNF39 *** **haplotypes on plasma HIV-1 viral load in the Han Chinese Population in Taiwan**

**Haplotypes (SNP1-SNP2/SNP1-SNP2)**	**Genotype frequency (no (%))**	**HIV-1 viral load (log10 copies/mL, mean (interquartile range))**^**a**^	***p*-value**^**b**^
Ht1 (GG/GG, n (%))	125 (73.5)	3.9 (2.6–6.4)	0.006*
Ht2 (GG/CA, n (%))	43 (25.3)	4.2 (2.6–5.8)	
Ht3 (CA/CA, n (%))	2 (1.2)		

The asterisk and bold, emphasizing statistical significance at *p* < 0.017 (0.05/3).

SNP1, rs3807032; SNP2, rs3807033.

^a^The HIV-1 viral load was measured in the peripheral blood when the patient was examined with the earliest HIV-1 antibody positive result. Any measurements taken after the initiation of antiretroviral therapy were not used in any analyses.

^b^*p*-values were obtained using the unpaired Student’s *t*-test.

**Figure 1:**
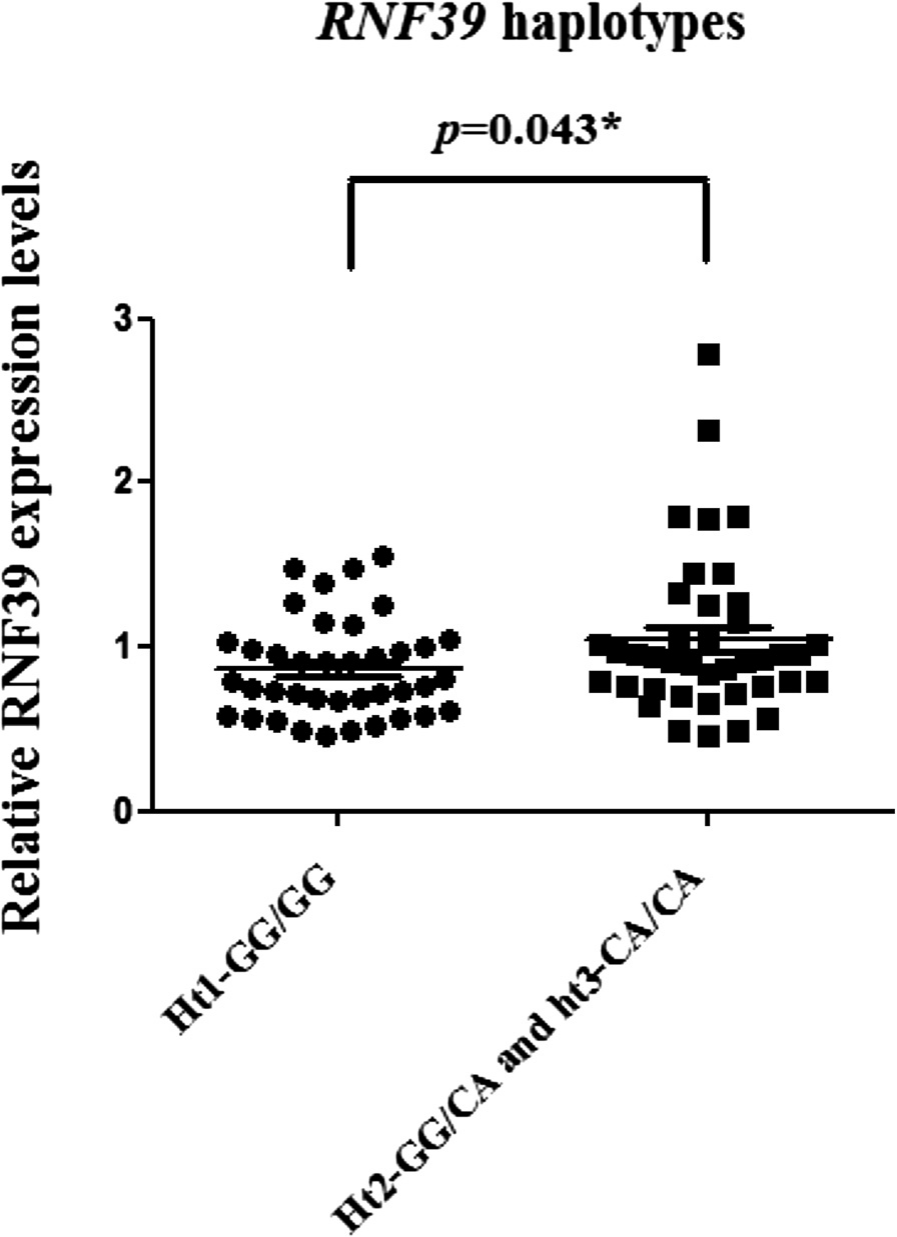
***RNF39 *mRNA expression levels in PBMCs between the *RNF39 *haplotypes.***RNF39* relative expression was detected by qPCR, and its expression in individuals with the ht1-GG/GG haplotype was compared with that in individuals with the ht2-GG/CA and ht3-CA/CA haplotypes. The relative expression levels were expressed as *RNF39* mRNA/HPRT mRNA ratio.

To investigate the correlation between *RNF39* haplotypes and its expression levels, we measured *RNF39* mRNA levels by real-time quantitative polymerase chain reaction (qPCR) in primary peripheral blood mononuclear cells from patients. As shown in Figure 1, *RNF39* expression level was lower in patients with the ht1-GG/GG haplotype than in patients with the ht2-GG/CA and ht3-CA/CA haplotypes (*p* = 0.043).

### RNF39 affects HIV-1 replication in HEK293T cells

**Figure 2:**
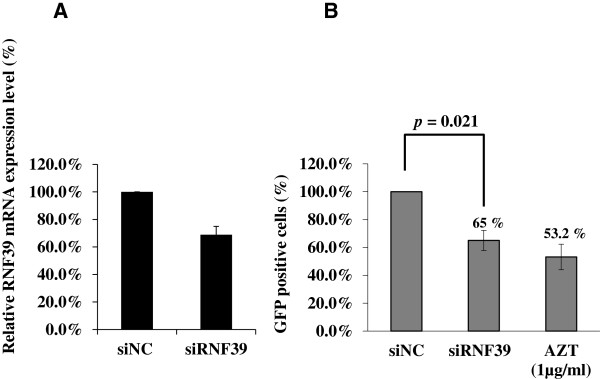
**RNF39 protein effects on HIV-1 replication in 293 T cells by using RNA interference assay. A**. RNF39 mRNA downregulation by RNA interference in 293 T cells. Relative *RNF39* mRNA was quantified by qPCR. Values are normalized to those of siNC-transfected cells. Data represent the mean ± SD of three independent experiments. **B**. Reduction of HIV-1 infection via RNF39 knockdown in 293 T cells. AZT-treated 293 T cells were used as a positive control for the reduction of HIV-1 infection.

To further investigate the role of RNF39 in HIV-1 replication, we used siRNA to knockdown *RNF39* expression in 293 T cells [15] (Figure 2A,B). HIV-1 GFP reporter virus NL4-△G/P-EGFP was employed as an indicator of viral replication. Cells were first transfected with siRNAs targeting *RNF39* (siRNF39) and the knockdown effect was assessed by qPCR. siRNA-transfected 293 T cells were then infected with NL4-3△G/P-EGFP. As shown in Figure 2B, a significant difference was observed in the number of GFP positive cells between the cells transfected with control siRNA (siNC) and those transfected with siRNF39. The number of GFP-expressing cells decreased to 65% (*p* = 0.021). Azidothymidine (AZT)-treated 293 T cells were used as positive controls; AZT treatment decreased HIV-1 infection to 53.2%.

**Figure 3:**
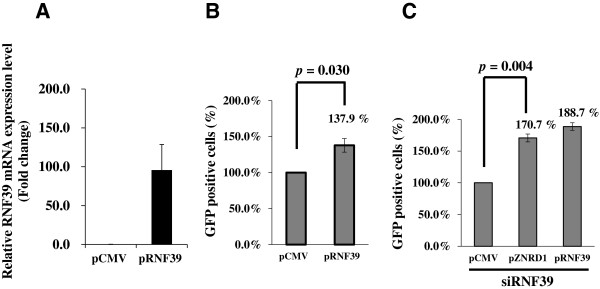
**RNF39 protein effects on HIV-1 replication in 293 T cells as evaluated by cDNA overexpression technique. A**. RNF39 mRNA overexpression in 293 T cells by using the RNF39 cDNA vector under CMV promoter control. *RNF39* relative mRNA expression was quantified by qPCR. Values are normalized to those of pCMV-transfected cells. Data represent the mean ± SD of three independent experiments. **B**. Enrichment of HIV-1 infection via RNF39 overexpression in 293 T cells. **C**. Enrichment of HIV-1 infection via RNF39 overexpression in siRNF39-pZNRD1- and siRNF39-pRNF39-transfected 293 T cells.

Next, RNF39 role in HIV-1 infection was also confirmed by overexpressing RNF39 cDNA (Figure 3A,B and C) in 293 T cells. 293 T cells were transiently transfected with RNF39 cDNA expression plasmid (pRNF39) and pCMV expression plasmid as a mock control (Figure 3A). Cells were then infected with NL4-3△G/P-EGFP. As shown in Figure 3B, the number of GFP positive cells infected with NL4-3△G/P-EGFP was increased in pRNF39-transfected cells compared with that in the control cells (137.9% increase, *p* = 0.030).

The cDNA overexpression method was further used to evaluate the effect of several proteins on HIV-1 infection after RNF39 knockdown by siRNA. siRNF39-treated 293 T cells were transfected with expression plasmids, including the pZNRD1, pRNF39, and pCMV plasmid. Cells were then infected with NL4-3△G/P-EGFP. As shown in Figure 3C, the number of GFP positive cells infected with NL4-3△G/P-EGFP increased after overexpression of RNF39 or ZNRD1 (188.7% for pRNF39 and 170.7% for pZNDR1, *p* = 0.004).

### RNF39 is also required for HIV-1 replication in Jurkat cells

**Figure 4:**
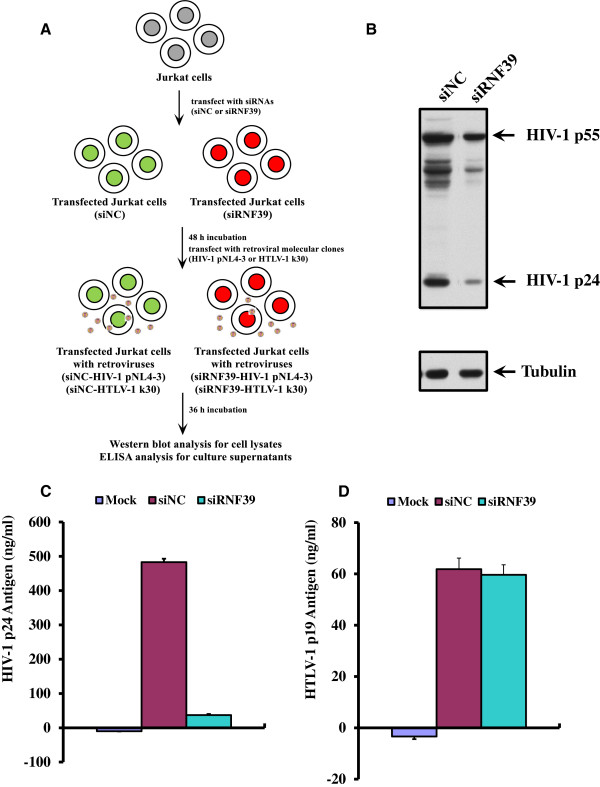
**RNF39 protein effects on HIV-1 replication in Jurkat cells as evaluated by RNA interference assay. A**. Flowchart depicting siRNA effect on the replication of retroviruses (HIV-1 molecular clone pNL4-3 and HTLV-1 molecular clone k30). **B**. Inhibition of HIV-1 replication by *RNF39* downregulation in Jurkat cells, measured using western blot analysis. **C**. Reduction of HIV-1 p24 antigen levels in culture supernatants from siRNF39-transfected Jurkat cells compared with culture supernatants from siNC-transfected cells, measured using HIV-1 p24 ELISA. **D**. No significant reduction of HTLV-1 p19 antigen levels was observed in culture supernatants from siRNAs-transfected Jurkat cells, measured using HTLV-1 p19 ELISA.

We also investigated the role of RNF39 in HIV-1 and HTLV-1 replication (Figure 4). Jurkat cells were transiently transfected with siRNAs. siRNA-treated Jurkat cells were then transfected with HIV-1 molecular clone pNL4-3 or HTLV-1 molecular clone k30 (Figure 4A). As shown in Figure 4B and Additional file 1: Figure S1, the expression of the HIV-1 p55 and p24 viral proteins was examined. The expression of the HIV-1 viral proteins was significantly decreased in the siRNF39-transfected Jurkat cells compared with that in the control siNC. Similarly, a reduction of HIV-1 p24 viral antigen levels was observed in the culture supernatants from siRNF39-transfected Jurkat cells compared with culture supernatants from the siNC-transfected cells (Figure 4C). No significant difference was observed in HTLV-1 p19 viral antigen levels in culture supernatants from siRNF39-transfected Jurkat cells (Figure 4D). Taken together, these results suggested that RNF39 is required for HIV-1 replication in Jurkat cells, whereas it does not affect HTLV-1 replication.

## Discussion

In the present study, we first showed that plasma HIV-1 viral loads are associated with *RNF39* genetic variants in patients infected with HIV-1. Furthermore, we found an association between *RNF39* haplotypes and HIV-1 viral loads. Patients with the *RNF39* ht1-GG/GG haplotype presented lower levels of HIV-1 viral load. Both SNPs are located in the 5′ regulatory region, which may affect nascent mRNA production or protein expression, thereby modulating HIV-1 production. Patients with the ht1-GG/GG haplotype exhibited lower levels of *RNF39* expression. Our results suggest that RNF39 expression knockdown inhibits HIV-1 expression, suggesting that RNF39 is required for viral replication. Genome-wide association studies (GWASs) revealed that human genetic variations, especially in genes located in the MHC region are involved in the control of HIV viral load, CD4 count, or the disease clinical course [11,16]. A few studies revealed that human genetic variations, especially genes located in the MHC region, close to *RNF39,* were associated with the control of HIV viral load, CD4 count, and/or the disease clinical course, but the results were still inconsistent [11,12,16,17]. *RNF39* SNPs are related to both disease progression and viral load [11,16]. *RNF39* SNPs have been associated with CD4 T-cell count, but not with disease course [12,17]. We showed that *RNF39* gene plays an important role in HIV replication and its genetic variants are associated with plasma viral loads in a Han Chinese cohort. Determinants for host control of infection, based on SNPs, still need to be confirmed among different populations, followed by detailed genomic and functional analyses of the associated region to verify putative SNPs.

By using RNA interference and complementation of RNF39 protein expression, we showed that RNF39 host cellular protein affects HIV-1 replication. These findings are in accordance with the identification of host cellular proteins that contribute to the HIV-1 life cycle [1,2]. Although Ballana *et al.* reported that RNF39 downregulation by siRNA did not impair HIV-1 replication in HeLa-derived cell lines [18], we used both lymphoid and non-lymphoid cell lines to demonstrate that RNF39 actually affects HIV-1 replication. RNF39 antiviral mechanism might be associated with the presence of the SPRY domain in RNF39 protein C-terminal [14]. This SPRY domain determines viral specificity and restriction potency, and is one of the major determinants for the host tropism of HIV-1 and related retroviruses [19-21]. To our knowledge, this is the first study to demonstrate a positive correlation between RNF39 and HIV-1 replication. Our results provide *in vitro* evidence regarding the role of RNF39 host cellular protein in HIV-1 replication and suggest that this protein may contribute to HIV-1 life cycle. Future studies in primary T cells and macrophages are warranted to confirm its function.

In conclusion, we provided further information that RNF39 is a host cellular protein required for HIV-1 replication *in vitro* and its genetic variants are associated with HIV-1 viral loads. Our findings emphasize the importance of studying individuals with a range of genetic backgrounds in HIV-1 infection research.

## Materials and methods

### Patients

This study was an observational study. The patients with HIV-1 were recruited from the Section of Infectious Diseases, Department of Internal Medicine, China Medical University Hospital, Taichung, Taiwan. Voluntary participants provided written informed consent and agreed to provide long-term follow-up clinical, epidemiological data, plasma, and peripheral blood mononuclear cells (PBMCs). Blood was collected by venipuncture. PBMCs were isolated by Ficoll-Histopaque (Sigma Aldrich, Saint Louis, MO, USA, 1077) density gradient centrifugation and frozen until use. HIV-1-antibody-positive individuals were recruited and HIV status was confirmed by quantitative HIV-1 RNA measurement (Roche, Basel, Switzerland; COBAS TaqMan HIV-1 assay v2.0).

HIV-1 viral loads were determined when the patient was examined with the earliest HIV-1 antibody positive result. Patients whose viral loads were measured after the initiation of antiretroviral treatment were excluded from the study. These patients with HIV-1 were (a) naïve for antiretroviral treatment, (b) belonged to the Han Chinese ethnic group, and (c) were willing to provide blood samples for genotyping. The study was approved by the Institutional Review Board of China Medical University Hospital. All participants read and signed informed consent documents.

### Detection of RNF39 genetic polymorphisms

*RNF39* SNPs were selected from the NCBI SNP database and HAPMAP website [13,22-24]. Selection criteria were a minor allele frequency >5% in the Han Chinese population and Hardy–Weinberg equilibrium (*p* > 0.05). A summary of *RNF39* gene SNPs information (location, position, rs number, and genotype) is presented in Table 2. Briefly, genomic DNA was extracted from PBMCs according to standard protocols (Genomic DNA kit, Qiagen, Limburg, Netherlands). SNPs were genotyped using a custom-designed VeraCode GoldenGate Genotyping Assay System (Illumina, San Diego, CA, USA) [25], and genotyping was performed as outlined on the Illumina website (http://www.illumina.com/).

### Real-time quantitative PCR

Total RNAs were isolated from PBMCs using the RNeasy Mini kit (Qiagen). *RNF39* mRNA relative levels from individuals were measured by qPCR and normalized to *HPRT* mRNA expression (Universal ProbeLibrary Assay Design Center, Roche). The relative expression levels were expressed as *RNF39* mRNA/HPRT mRNA ratio (Figure 1). For human genes, primers and DNA probes were commercially designed and purchased (*RNF39* gene, forward primer: 5′-agactgacagccgacctga-3′ and reverse primer: 5′-ctggtggggccagttgta-3′, probe 70 from Universal ProbeLibrary).

### Statistical analyses

Genotypes were obtained by direct count, followed by allele frequency calculations (Table 2). The unpaired Student’s *t*-test was used for comparison between groups (Tables 2 and 3). Lewontin’s D’ measure was used to estimate the intermarker coefficient of linkage disequilibrium (LD) of our patients with HIV-1 using HAPLOVIEW software [24]. The LD confidence interval was estimated using a resampling procedure and was used to construct the haplotype blocks [26]. Haplotypes were inferred from unphased genotype data by using the Bayesian statistical method available in the program Phase 2.1 [27-29]. All statistical analysis was performed using SPSS (v12.0; IBM, Chicago, IL, USA) for Windows, and graphs were generated using GraphPad Prism version 5.01 for Windows (GraphPad Software, San Diego, California, USA).

### Cells and recombinant virus

293 T cells (ATCC, Manassas, VA, USA; accession no. CRL-11268) were cultured in Dulbecco’s Modified Eagle’s Medium (DMEM) supplemented with 10% fetal bovine serum (FBS) and 100 U/mL penicillin, 100 U/mL streptomycin, and 2 mM L-glutamine (Gibco). Jurkat cells (ATCC; accession number TIB-152) were grown in RPMI-1640 medium supplemented with 10% FBS and 100 U/mL penicillin, 100 U/mL streptomycin, and 2 mM L-glutamine.

HIV-1 pseudotyped virus NL4-3△G/P-EGFP was produced from 293 T cells as previously described [15]. Briefly, cells were cotransfected with three package vectors including pCMV△R8.2 (the package vector), pMD.G (VSV-G expression vector), and pNL4-3:△G/P-EGFP (HIV-1 molecular clone NL4-3 strain with *EGFP* reporter gene, without viral *gag* and *pol* genes). After 48 h of incubation, viruses were collected from the culture supernatant and titrated on 293 T cells based on GFP expression. Cells were infected with NL4-3△G/P-EGFP (multiplicity of infection (moi) = 5) in the presence of 6 μg/mL polybrene (Sigma).

The HIV-1 (pNL4-3) and HTLV-1 k30 molecular clones and the viral Tax gene clone were obtained from the NIH AIDS Research and Reference Reagent Program [30-32]. All media were supplemented with 100 U/mL penicillin, 100 U/mL streptomycin, and 2 mM L-glutamine.

### Drug and short interfering RNAs

AZT was commercially obtained (Sigma). Short Interfering RNAs (siRNAs) targeting transcripts for *RNF39* (siRNF39: GACUGAGACUCUGGUUGAAGAGAGA) and the non-targeting siRNA control (siNC) were purchased from Invitrogen (Carlsbad, CA, USA).

### siRNA or cDNA plasmid transfection

For 293 T cells, cells (2 × 10^5^) were seeded in 24-well plates and cultured for 24 h. siRNAs or cDNA plasmid targeting *RNF39* (NM_025236) (Figure 2A,B and Figure 3A,B and C) were mixed with Roche X-tremeGENE Transfection Reagent (Roche) in serum-free medium (OPTI-MEM I, Invitrogen) following the manufacturer’s recommendations. siRNA or cDNA plasmid-transfected 293 T cells were then infected with NL4-3△G/P-EGFP (moi = 5) in the presence of 6 μg/mL polybrene (Sigma) for 36 h. After infection, cells were used for the quantification of EGFP expression by flow cytometry.

For Jurkat cells, cells (2 × 10^5^) were seeded in 24-well plates and cultured for 24 h. siRNAs targeting *RNF39* (NM_025236) (Figure 4) were transfected using Amaxa Nucleofector Technology (Lonza, Basel, Switzerland) following the manufacturer’s recommendations. Cells were then recovered and seeded in prewarmed 24-well plates. After 48 h incubation, Jurkat cells were transfected with pNL4-3 or HTLV-1 molecular clone k30 and incubated for another 36 h. Cells were then used for HIV-1 western blot analysis (Figure 4B), HIV-1 p24 antigen enzyme-linked immunosorbent assay (ELISA) (Figure 4C), and HTLV-1 p19 antigen ELISA (Figure 4D).

### Reagents and western blot

The anti-HIV-1 antibody was obtained from the NIH AIDS Research and Reference Reagent Program. The anti-beta tubulin antibody was obtained from GeneTex (GeneTex, Irvine, CA, USA; GTX101279). The HTLV-1 p19 ELISA kit and anti-p19 antibody were from Zeptometrix (Buffalo, NY, USA). The experimental protocol for western blot was previously described [33,34]. Briefly, cells were harvested, washed, and lysed in lysis buffer (50 mM Tris–HCl [pH 7.5], 150 mM NaCl, 5 mM EDTA, 1% Triton X-100, 0.1% SDS) supplemented with protease inhibitor cocktail (Roche). The lysates were resolved by 12% SDS-PAGE and transferred to polyvinylidene fluoride membranes (Millipore, Billerica, MA, USA). The membrane was incubated with primary antibodies overnight at 4°C, and then incubated with alkaline phosphatase-conjugated secondary antibodies (Sigma). Signals were visualized using chemiluminescence following the manufacturer’s protocol (Chemicon, Billerica, MA, USA). Band intensities were quantified using ImageJ software.

## Competing interests

The authors declare that they have no competing interests.

## Authors’ contributions

YJL, CYC, KTJ, XL, and FJT conceived and designed the experiments. CYC, THL, CCL, SMH, and CWL performed the experiments. HT, WKC, and JHC analyzed the data. CYC, XL, JHW, CHH, MWH, and TJH contributed reagents/materials/analysis tools. YJL, XL, and TJH wrote the manuscript. All the authors have read and approved the final manuscript.

## Supplementary Material

Additional file 1: Figure S1 Inhibition of HIV-1 replication by *RNF39* RNA interference-mediated silencing in Jurkat cells. Upper diagram: relative HIV-1 p55 viral protein levels were monitored by band intensities quantified using ImageJ software. Lower diagram: relative HIV-1 p24 viral protein levels were monitored by band intensities quantified using ImageJ sofware. Relative HIV-1 viral protein levels were calculated as a ratio of the band intensities of siRNF39-treated cells to siNC-treated cells. The western blot data was shown in Figure 4B and these results represents mean ± for three independent experiments. Click here for file
