# Supplementary material for: Ring finger protein 39 genetic variants associate with HIV-1 plasma viral loads and its replication in cell culture
Source: Cell Biosci. 2014 Aug 5;4:40. doi: 10.1186/2045-3701-4-40 (PMC4131809; doi:10.1186/2045-3701-4-40)
Supplement: Additional file 1: Figure S1 — Inhibition of HIV-1 replication by RNF39 RNA interference-mediated silencing in Jurkat cells. Upper diagram: relative HIV-1 p55 viral protein levels were monitored by band intensities quantified using ImageJ software. Lower diagram: relative HIV-1 p24 viral protein levels were monitored by band intensities quantified using ImageJ sofware. Relative HIV-1 viral protein levels were calculated as a ratio of the band intensities of siRNF39-treated cells to siNC-treated cells. The western blot data was shown in Figure 4B and these results represents mean ± for three independent experiments. [file 2045-3701-4-40-S1.ppt]

## Slide 1
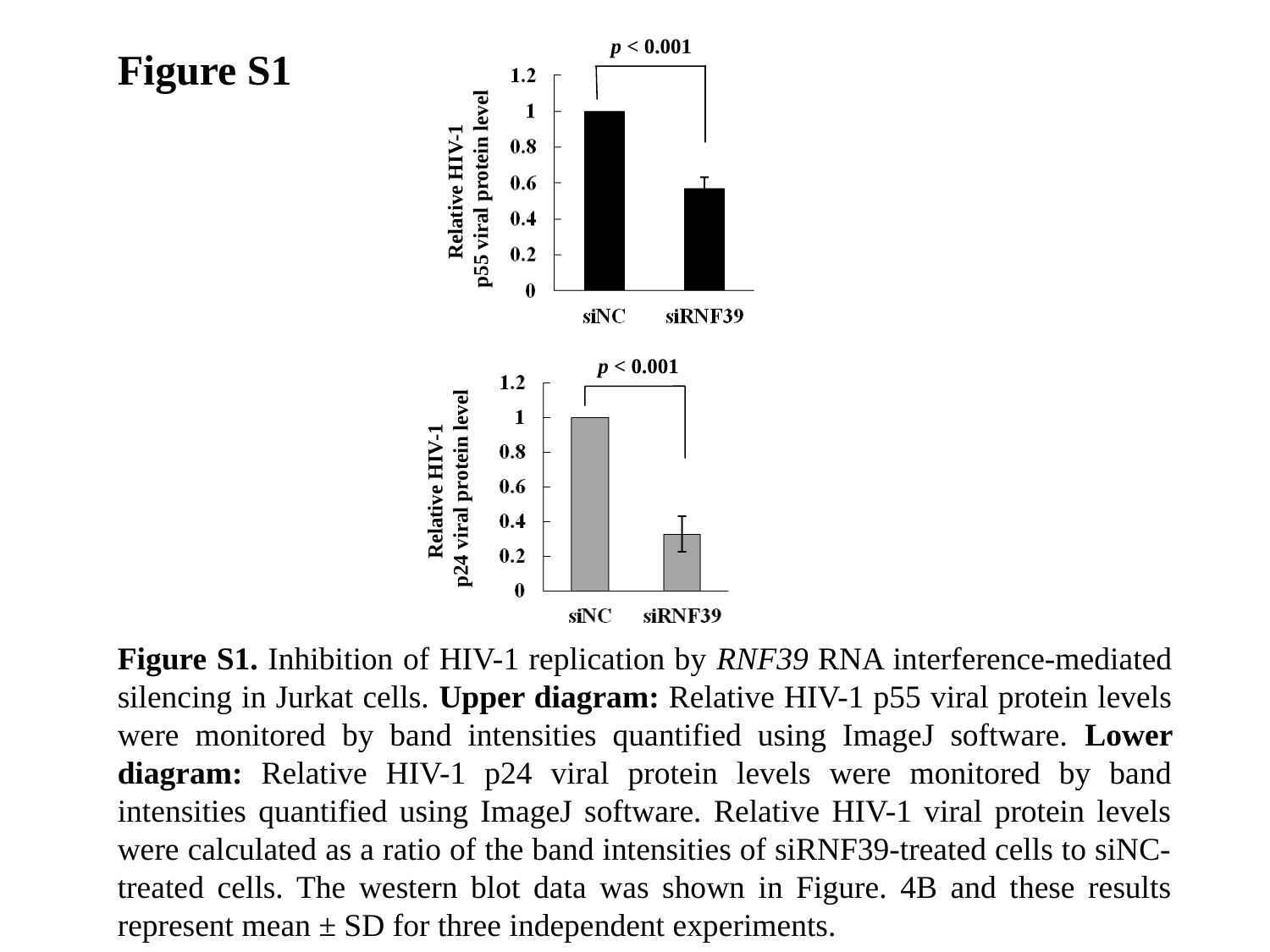

p < 0.001
Figure S1
Relative HIV-1
p55 viral protein level
p < 0.001
Relative HIV-1
p24 viral protein level
Figure S1. Inhibition of HIV-1 replication by RNF39 RNA interference-mediated silencing in Jurkat cells. Upper diagram: Relative HIV-1 p55 viral protein levels were monitored by band intensities quantified using ImageJ software. Lower diagram: Relative HIV-1 p24 viral protein levels were monitored by band intensities quantified using ImageJ software. Relative HIV-1 viral protein levels were calculated as a ratio of the band intensities of siRNF39-treated cells to siNC-treated cells. The western blot data was shown in Figure. 4B and these results represent mean ± SD for three independent experiments.
